# Fatigue life of 3D-printed porous titanium dental implants predicted by validated finite element simulations

**DOI:** 10.3389/fbioe.2023.1240125

**Published:** 2023-08-10

**Authors:** Antoine Vautrin, Jensen Aw, Ed Attenborough, Peter Varga

**Affiliations:** ^1^ AO Research Institute Davos, Davos, Switzerland; ^2^ Graduate School for Cellular and Biomedical Sciences, University of Bern, Bern, Switzerland; ^3^ Attenborough Dental Laboratories Ltd, Nottingham, United Kingdom

**Keywords:** dental implant, fatigue, porous titanium, additive manufacturing, finite element analysis, micro-CT

## Abstract

**Introduction:** Porous dental implants represent a promising strategy to reduce failure rate by favoring osseointegration or delivering drugs locally. Incorporating porous features weakens the mechanical capacity of an implant, but sufficient fatigue strength must be ensured as regulated in the ISO 14801 standard. Experimental fatigue testing is a costly and time-intensive part of the implant development process that could be accelerated with validated computer simulations. This study aimed at developing, calibrating, and validating a numerical workflow to predict fatigue strength on six porous configurations of a simplified implant geometry.

**Methods:** Mechanical testing was performed on 3D-printed titanium samples to establish a direct link between endurance limit (i.e., infinite fatigue life) and monotonic load to failure, and a finite element model was developed and calibrated to predict the latter. The tool was then validated by predicting the fatigue life of a given porous configuration.

**Results:** The normalized endurance limit (10% of the ultimate load) was the same for all six porous designs, indicating that monotonic testing was a good surrogate for endurance limit. The geometry input of the simulations influenced greatly their accuracy. Utilizing the as-designed model resulted in the highest prediction error (23%) and low correlation between the estimated and experimental loads to failure (R^2^ = 0.65). The prediction error was smaller when utilizing specimen geometry based on micro computed tomography scans (14%) or design models adjusted to match the printed porosity (8%).

**Discussion:** The validated numerical workflow presented in this study could therefore be used to quantitatively predict the fatigue life of a porous implant, provided that the effect of manufacturing on implant geometry is accounted for.

## 1 Introduction

Dental implants must sustain the mastication loads exerted on natural teeth. These repetitive forces can cause implant loosening or failure that require extraction and replacement of the defective implant. Peri-implantitis affects up to 20% of patients (Mombelli et al., 2012) and is a is a notable cause of implant failure. Peri-implantitis is characterized by an infection of the gingival soft tissue that can then propagate to bone and cause its resorption, which in turn increases the likelihood of mechanical implant failure. The long-term stability of dental implants is strongly determined by the osseointegration process. Formation of bone at the implant’s surface results in establishing a load transfer mechanism between the implant and the surrounding bone (Li et al., 2020). Incorporating porosities in a dental implant increases the contact surface area with bone and thus represents a promising strategy to improve long-term stability. Bony ingrowth inside pores creates a mechanical interlocking between the two structures (Pałka and Pokrowiecki, 2018). Alternatively, the failure risk of dental implants can be reduced by using porous implants as drug-delivery devices: their pores can be loaded with antibiotics to treat locally infections such as peri-implantitis (De Cremer et al., 2017).

The production of complex implant geometries such as porous structures was enabled by the development of metal additive manufacturing (AM) (Hao et al., 2016). Titanium has been the predominantly used material for dental implants due to its advantageous properties in terms of biocompatibility, resistance to corrosion and osteoinductivity (Haugen and Chen, 2022). Titanium has also been used to develop porous scaffolds for dental and orthopaedic applications (Lv et al., 2021). The introduction of porosities in implants reduces mechanical strength relative to their solid counterparts and their mechanical behavior depends not only on the porosity level but also on the pore geometry (Maconachie et al., 2019; Chen et al., 2020). The mechanical properties of a wide range of porous configurations have been determined by testing scaffolds under both monotonic (Soro et al., 2019; Xiong et al., 2020) and fatigue loads (Li et al., 2016; Dallago et al., 2018; Lietaert, 2018).

Dental implants must comply with the ISO 14801 standard to ensure sufficient mechanical strength. This standard describes the mechanical testing procedure to determine endurance limit, i.e., the load level at which the implant has an infinite lifetime, i.e., it will not fail during its intended usage period. The prescribed bending-compression fatigue loading mode mimics the cyclically applied forces caused by mastication. However, a high number of samples (N ≥ 15) per design are required and the experiments are time-consuming with a single test lasting up to 4 days. Although several studies have investigated the failure of conventional implants in this testing configuration (Shemtov-Yona and Rittel, 2014; Song et al., 2017; Rojo et al., 2018; Velasco-Ortega et al., 2019), only a few have focused on porous implants (Wang et al., 2019; Vanmunster et al., 2022; Lovera-Prado et al., 2023). Most studies investigating porous dental implants utilized monotonic tests in a variety of loading modes such as uniaxial compression, bending-compression, three-point bending and pull-out (Chakraborty et al., 2020; Huang et al., 2020). These tests have the advantage of being quicker than fatigue testing while allowing a qualitative comparison of different designs but may not be relevant for the fatigue failure required by the ISO 14801 standard.

The design process of implants is an iterative process requiring several prototypes. However, testing multiple porous configurations according to ISO 14801 is costly and time consuming and hence the appeal of developing alternative methods to quickly compare the fatigue life of multiple designs has arisen. Finite element (FE) modeling is a promising approach that meets this criterion and has been widely used to simulate the mechanical behavior of biomedical (Taylor and Prendergast, 2015; Lewis et al., 2021; Panagiotopoulou et al., 2021) and dental (Trivedi, 2014; Reddy et al., 2019) implants. Previous studies have utilized FE simulations to model ISO 14801 testing of conventional dental implants (Prados-Privado et al., 2018; Lee et al., 2021), but only a single study has modeled the ISO testing of a porous dental implant with a single pore design (Wang et al., 2019). The design process requires the evaluation of several configuration. Although the effect of unit cell geometry and porosity on the uniaxial fatigue strength of porous scaffolds has been studied both *in vitro* and *in silico* (Zheng et al., 2018; Maconachie et al., 2019; Benedetti et al., 2021), the impact of porous configuration has not been investigated for porous dental implants under bending-compression prescribed by the ISO 14801 standard. Therefore, the aim of this work is to develop, calibrate, and validate an FE-based tool to predict the fatigue life of 3D-printed porous titanium specimens mimicking dental implants.

## 2 Materials and methods

**FIGURE 1:**
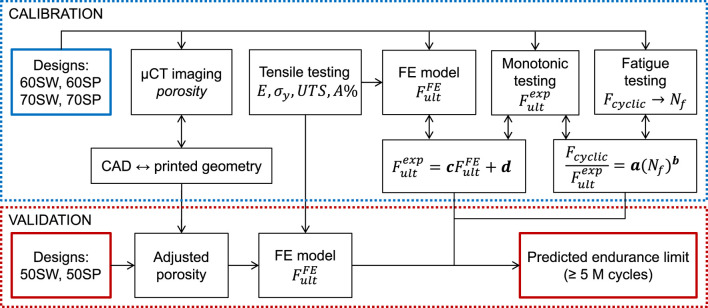
FE-based workflow for fatigue life prediction consisting of two loops. The three constitutive relationships of the model (between fatigue loading and number of cycles to failure, between experimental and simulated ultimate monotonic load, and between CAD and printed geometries) were determined in the calibration loop by combining µCT imaging, mechanical testing, and FE modeling. In the validation loop, the endurance limit of a separate set of designs was predicted using the calibrated models to assess the accuracy of the workflow.

We have established and validated a computational workflow to predict the endurance limit of porous specimens directly from their CAD models (Figure 1). Specifically, the tool uses CAD-based FE models to predict the monotonic failure load for various pore types and porosities and, consecutively, the fatigue life (i.e., endurance limit) based on empirically established relationships.

Calibration of the tool was performed by establishing three relationships. First, the differences between planned CAD-based and printed geometries were evaluated with micro computed tomography (µCT) image analysis and conversion rules were determined. Second, experimental testing was performed to establish the link between fatigue and monotonic behaviors. Lastly, FE simulations were performed to predict the ultimate load and correlate it with the experimental ultimate load. Calibration was achieved in a series of experiments involving four specimen groups of different pore types and porosities. The workflow was then validated on two other specimen groups with different porosities.

### 2.1 Sample design and manufacturing

**FIGURE 2:**
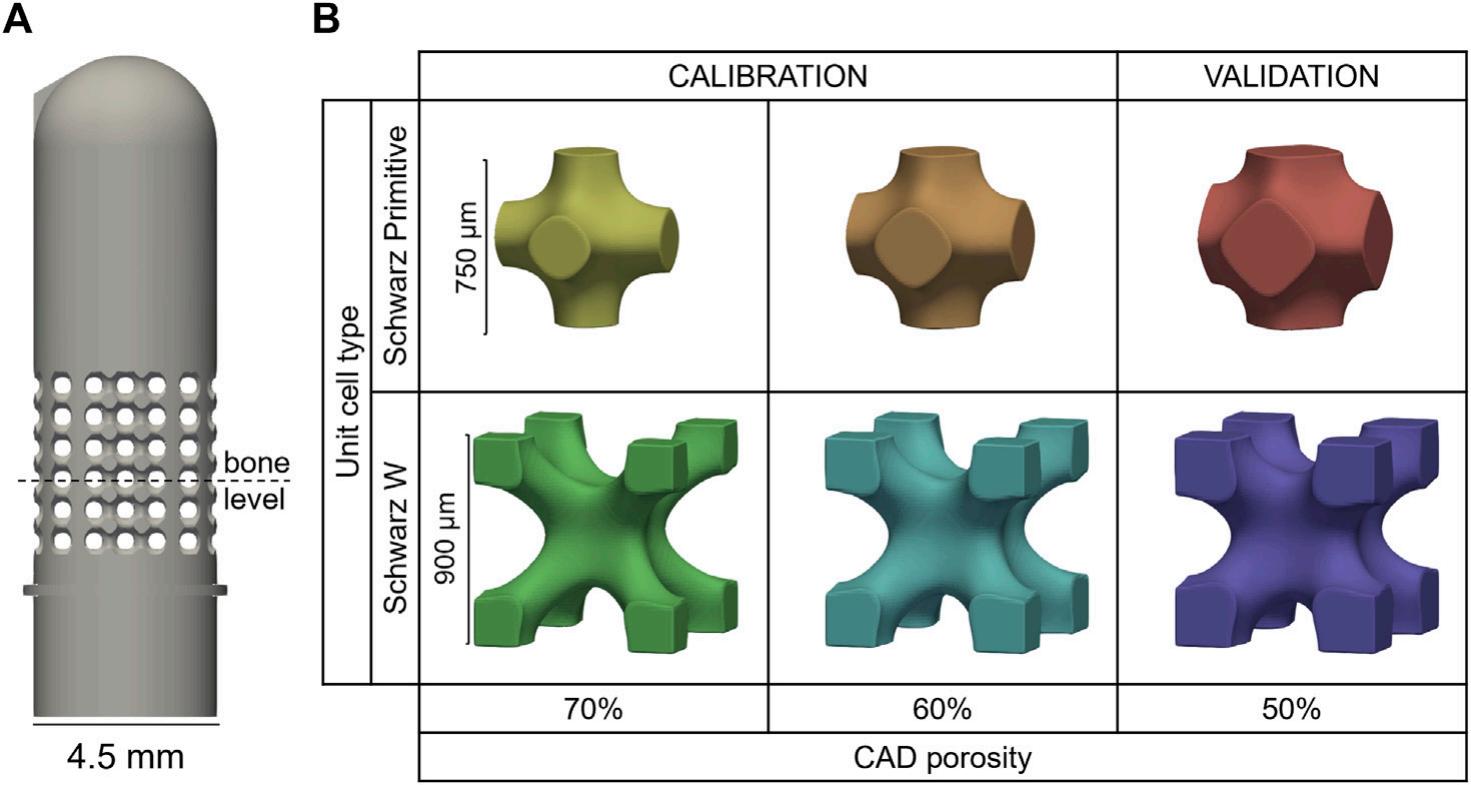
**(A)** Porous sample geometry and **(B)** unit cell models of the six porous configurations manufactured by 3D printing.

Simplified test samples mimicking dental implants were designed as 4.5 mm-diameter cylinders that comply with the ISO 14801 requirements, i.e., the distance between the embedding plane and the center of the hemispherical tip being 11 mm (BS EN ISO 14801 - 2016). In the hypothesis of a drug-delivery implant, the porous region should be placed close to the soft tissue, i.e., where an infection is most likely to happen. Hence, a fully porous section of 4.5 mm height introduced around nominal bone level (Figure 2A). Two unit cell geometries (skeletal Schwarz Primitive (SP) and Schwarz W (SW) (Zheng et al., 2018)) and three porosity levels (50%, 60% and 70%) were combined to generate six designs: 50SP, 60SP, 70SP, 50SW, 60SW and 70SW (Figure 2B). The bulk part of the design was generated with Solidworks 2021 (Dassault Systèmes Simulia, Unites States) and then imported in Simpleware Scan IP (M-2017.06, Synopsys Inc., Unites States) to generate the porous section. The length of the unit cell was selected to fit an integer number of cells within the height of the pore region (4.5 mm). Based on the findings of Zheng et al. showing that for equal unit cell lengths the pores of SP are two times larger and its struts 50% wider than the ones of SW (Zheng et al., 2018), a larger cell size was assigned to SW in order to reduce this difference (Figure 2B). Two additional features were added: a ridge at embedding level and a protuberance at the top of the implant to ensure consistent height and rotation, respectively.

A total number of 168 samples (28 per design) were additively manufactured via selective laser melting with Ti6Al4V powder (EOSINT M270, with printing parameters: 170 W laser power, 1,250 mm/s scanning speed and 0.03 mm layer thickness). The predictive capability of the developed tool was assessed by dividing the into two groups: a calibration (60SP, 60SW, 70SP, 70SW) and a validation (50SP, 50SW) set (Figure 2B).

### 2.2 µCT imaging

Four samples of each design were scanned by µCT with a voxel size of 10.5 µm (vivaCT 80, Scanco Medical AG, Switzerland) using a tube energy of 70 kV and a current of 0.114 mA. The implant was segmented in the µCT images with a global threshold and exported as binary masks using Amira 3D 2021.2 (Thermo Fisher Scientific, Unites States). The effective printed porosity was computed in a 3 mm × 3 mm x 3 mm cubic ROI located in the center of the porous section with the BoneJ plugin (ImageJ 1.53d) (Doube et al., 2010; Schneider et al., 2012). The relationship between designed and printed porosities was determined for each unit cell assuming dependence of printability on cell geometry (Maconachie et al., 2019). This provided a pore shape specific correction factor that was utilized in the computational workflow. To compensate for printing inaccuracies, adjusted CAD models were generated by setting the porosity level of a CAD model to the one evaluated from the µCT images.

### 2.3 Mechanical testing

**FIGURE 3:**
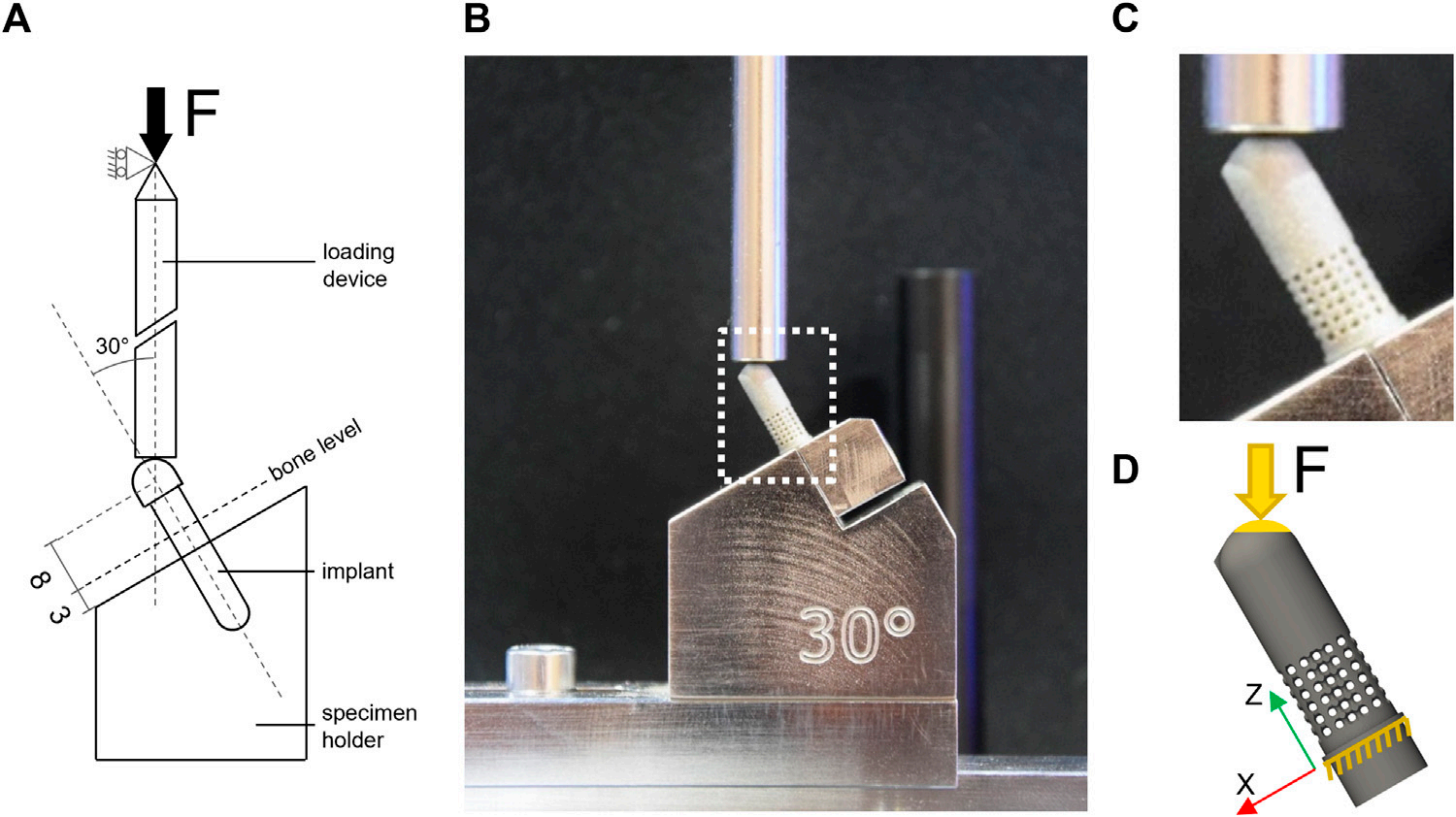
Experimental and computational testing **(A)** description of the test setup based on BS EN ISO 14801; **(B)** experimental test setup used for monotonic and fatigue testing; **(C)** zoom-in view on the 60SP sample mounted in the testing machine; **(D)** representation of the FE model of the 60SP CAD geometry and boundary conditions replicating the experimental setup: blocked displacement under the embedding plane and load applied to the top of the sample at 30° off-axis to the contact surface.

The porous samples were tested in accordance with the ISO 14801 standard (Figure 3A) under two loading types, monotonic and fatigue. The standard prescribes loading under bending compression at 30° off-axis. Additional uniaxial tensile testing was performed on dog bone-shaped samples to determine the mechanical properties of the 3D-printed material.

#### 2.3.1 Monotonic testing

Four samples per design (24 samples in total, including one µCT-scanned specimen/design) were tested monotonically in ISO 14801 configuration under a quasi-static displacement rate of 0.4 mm/min using an Instron 5866 testing machine (Instron, Unites States) equipped with a load cell having 10 kN capacity. The force-displacement curve was recorded and the ultimate load ( $F_{ult}^{\exp}$ ) was determined as the maximum force reached throughout the test.

#### 2.3.2 Fatigue testing

Fatigue loading was performed according to ISO 14801. The applied load was set to oscillate between 10% and 100% of the maximum load $F_{cyclic}$ at a frequency of 15 Hz (DYNA5dent, DYNA-MESS Prüfsysteme GmbH, Germany). For each design, six load levels were defined as fractions of $F_{ult}$ and rounded to the nearest 10 N: 50%, 35%, 25%, 20%, 15% and 10%. In total, 144 samples were tested in fatigue (24 samples/design, including three µCT-scanned specimen/design, one for each of the highest load levels). Four samples of the same design were tested at each of the six load levels. Sample survival was defined in accordance with ISO 14801 as enduring five million loading cycles. In case the sample failed earlier, the number of cycles to failure ( $N_{f}$ ) was recorded.

Our workflow assumed that the relationship between the normalized fatigue load $F_{cyclic}/F_{ult}^{\exp}$ and $N_{f}$ was the same for all designs, i.e., that $F_{ult}^{\exp}$ was directly proportional to a design’s fatigue behavior. The fatigue failures data for all designs was fitted with the following power law:

$$F_{cyclic}/F_{ult}^{\exp}=a{\left(N_{f}\right)}^{b}$$ (1)

with *a* and *b* being constants that were calibrated based on the experimental results of the calibration sample set. Moreover, it was assumed that the endurance limit could be estimated with this equation by calculating the fatigue load for $N_{f}=5.10^{6}$ cycles.

#### 2.3.3 Uniaxial tensile testing

**FIGURE 4:**
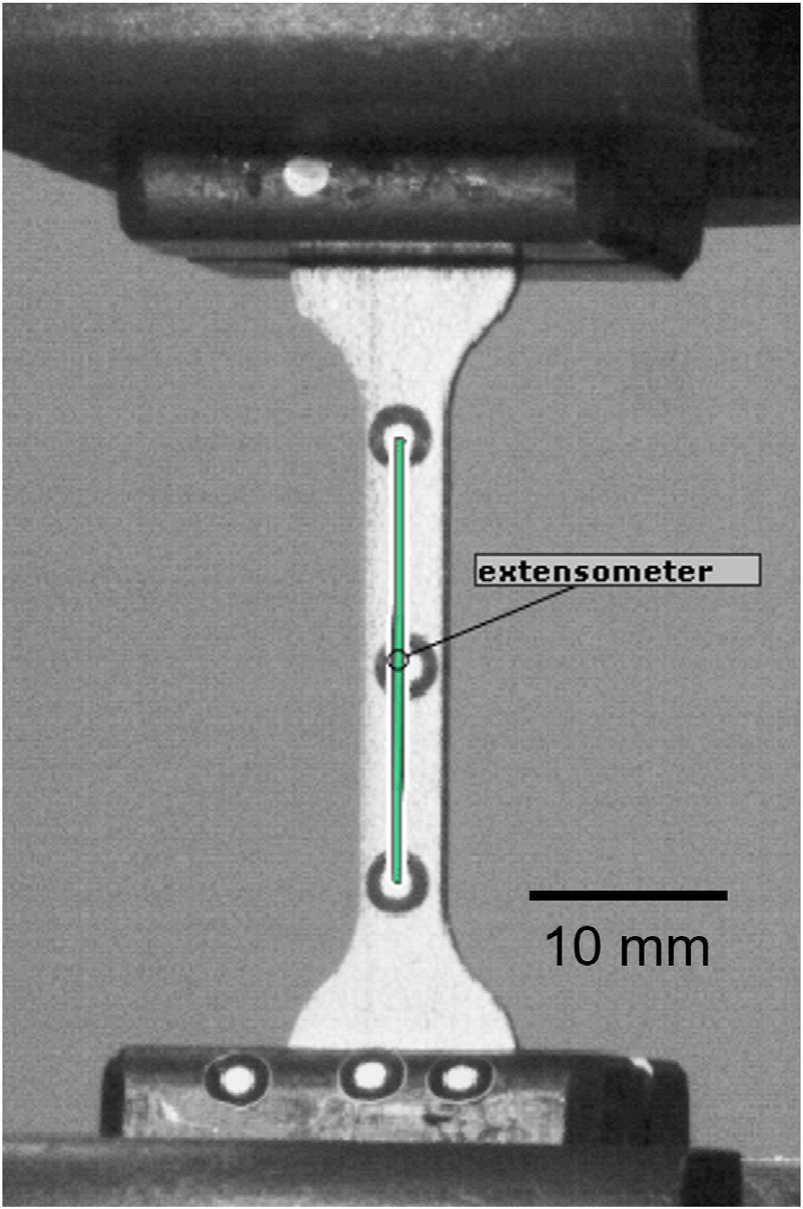
Tensile testing setup captured by the Aramis tracking system. The green line corresponds to the landmarks-based extensometer.

The elastic and plastic material properties of the 3D printed Ti6Al4V material were determined via uniaxial tensile testing based on the ISO 6892 standard (Instron 5866, Instron, Unites States) using six standard dog bone-shaped samples (cross section: 4 mm × 1.5 mm, total length: 70 mm). Stress was computed by dividing the machine reaction force measured by the 10 kN load cell (Instron) by the central cross-section, which was measured for each sample with a digital caliper. A stereographic optical tracking system (Aramis SRX, GOM GmbH, Germany) was used to track the displacement of two markers placed at both extremities of the middle section (Figure 4). These two points act as a digital extensometer from which longitudinal strain was assessed by dividing the change in distance with the original length. The elastic modulus (E) was computed as the slope of the linear part of the stress-strain curve, i.e., between 0 and 400 MPa, using linear regression analysis. The 0.2% plastic strain offset method was used to determine yield stress (σ_y_). The ultimate tensile strength (UTS) and elongation at break (A%) were determined as the maximum stress and the strain values, respectively.

### 2.4 FE simulations

#### 2.4.1 Model geometry and material properties

**FIGURE 5:**
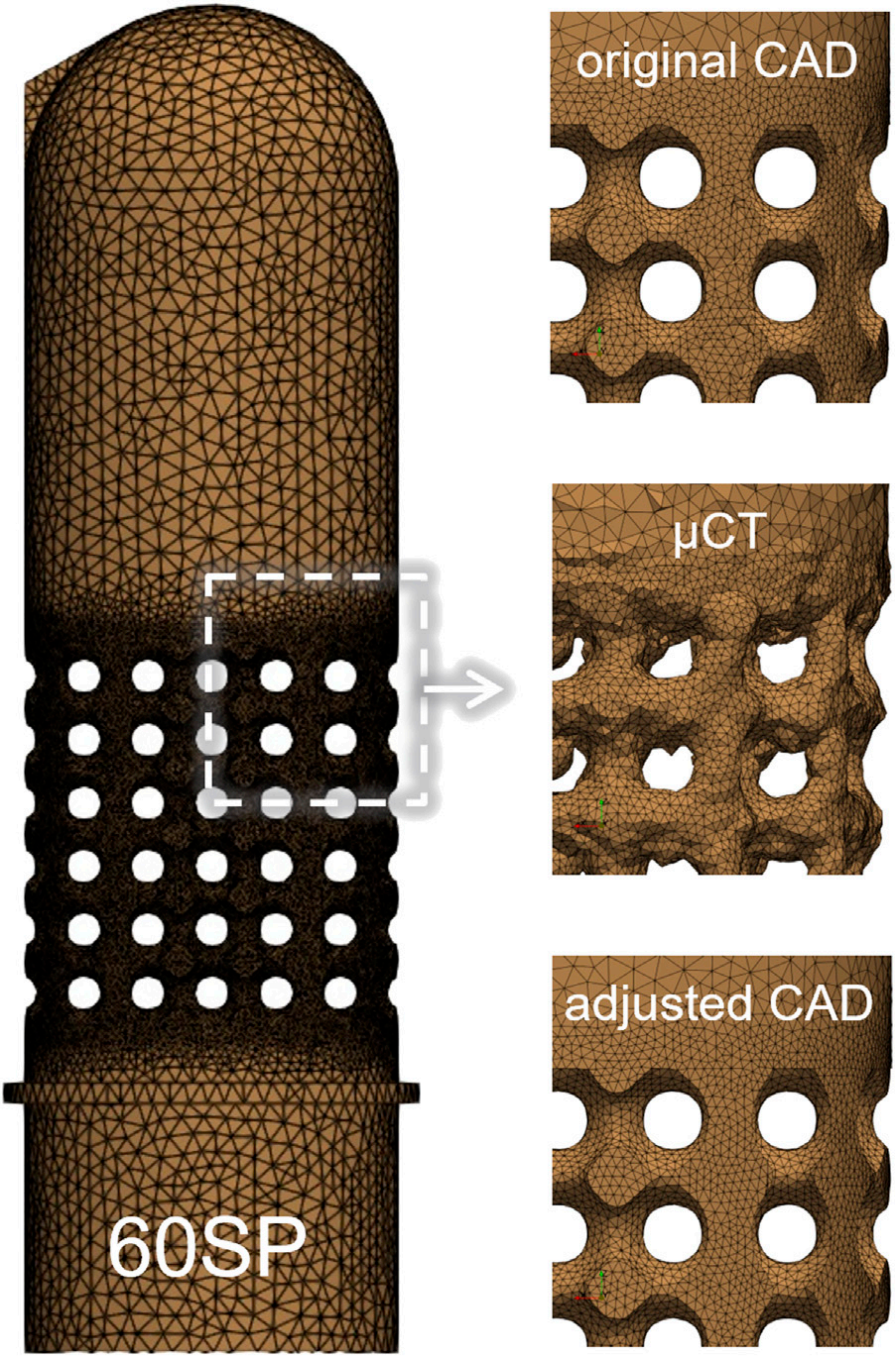
FE mesh of the 60SP design (left) with zoomed sections of the porous part for the tree types of geometry used in the simulations: original CAD, µCT-based and adjusted CAD (right).

Linear elastic FE models were built for each sample design. To investigate the effect of printing inaccuracies and to define an approach to compensate for these, three different strategies were implemented for defining the model geometries from three different data sources and types: the original CAD model, and the masks generated from the segmented µCT image and the adjusted CAD model matching the printed porosity level (*cf.* section 2.2) (Figure 5). These models were meshed in Simpleware with quadratic tetrahedral elements (C3D10). After performing a mesh convergence study, the element edge length was set to the range [0.15 mm; 0.4 mm] for the whole model and was refined to 0.1 mm for the porous region, with a resulting number of elements between 339,000 and 799,000.

An elasto-plastic material behavior law was assigned to titanium with linear elasticity and isotropic plasticity. The material properties determined by uniaxial tensile testing (E = 104.8 GPa, σ_y_ = 820.6 MPa, UTS = 914.6 MPa, A% = 4.41%, see section 3.2.3) were implemented here and a Poisson’s coefficient of 0.3 was used.

#### 2.4.2 Loading and boundary conditions

The loading conditions were set to mimic the experimental conditions reproducing the ISO standard. The displacement of all nodes located below the embedding plane was constrained in all directions and loading was applied at 30° off-axis direction (Figure 3D). A single concentrated force was applied to a virtual node located at the loading point that was kinematically coupled to all surface nodes of the hemispherical head within a radius of 1 mm to apply the load evenly on the contact surface.

#### 2.4.3 Ultimate load determination

The FE simulations aimed to determine the ultimate state and the corresponding force level ( $F_{ult}^{FE}$ ). Six models were simulated for each design: the original CAD-based, the adjusted CAD-based and four µCT-based models representing the four scanned samples. The analyses were performed with the standard solver of Abaqus 2021 (Dassault Systèmes, Simulia, Unites States) by applying a load magnitude of 1000 N to ensure that the ultimate state was reached. The FE simulation results were post-processed by extracting the load-displacement curves at the load application node. $F_{ult}^{FE}$ was determined on the force-displacement curves using the secant method using a displacement offset. The plastic displacement ( $u^{pl}$ ) was computed as follows:

$$u^{pl}=u-F/k$$ (2)

with u being the displacement of the node where the force was applied in mm, F the applied load in N and k the elastic stiffness of the sample in N/mm. Here, it was assumed that failure occurred at the same plastic displacement offset $u_{ult}^{pl}$ for all designs. This threshold value was identified in a parametric sub-study providing the strongest correlation between $F_{ult}^{FE}$ of the µCT-based models and $F_{ult}^{\exp}$ for the calibration set.

#### 2.4.4 Endurance limit prediction and model validation

The FE-based ultimate load $F_{ult}^{FE}$ that then was correlated with the experimental ultimate force data with the following equation:

$$F_{ult}^{\exp}=c∙F_{ult}^{FE}+d$$ (3)

with c and d being pore type specific constants. The FE-based fatigue life prediction expressed by combining the relationships between the results of fatigue and monotonic testing (Eq. 1) as well as between monotonic testing and FE simulations (Eq. 3) is as follows:

$$F_{cyclic}=a\left(c∙F_{ult}^{FE}+d\right){\left(N_{f}\right)}^{b}$$ (4)

Three parameters of this equation ( c , d and $u_{ult}^{pl}$ ) were calibrated with the experiments and simulations performed on the four designs of the calibration set (70SW, 70SP, 60SW, 60SP) (Figure 1). Finally, model validation was performed for each geometry type (CAD-based, µCT-based and adjusted CAD-based) by predicting the ultimate loads of the designs of the validation set (50SW, 50SP) via FE simulations. The endurance limits were computed according to Eq. 4 and compared with the corresponding experimental results to benchmark the different geometry types.

### 2.5 Statistical analysis

The predictive capability of each model type was assessed using linear regression analysis on the calibration dataset to compare the FE-based and experimental ultimate loads. The Pearsons’s correlation coefficient (R^2^) values and *p*-values were used as measures of predictive performance and statistical significance, respectively. The significance level was set at 0.05.

## 3 Results

### 3.1 µCT imaging

**FIGURE 6:**
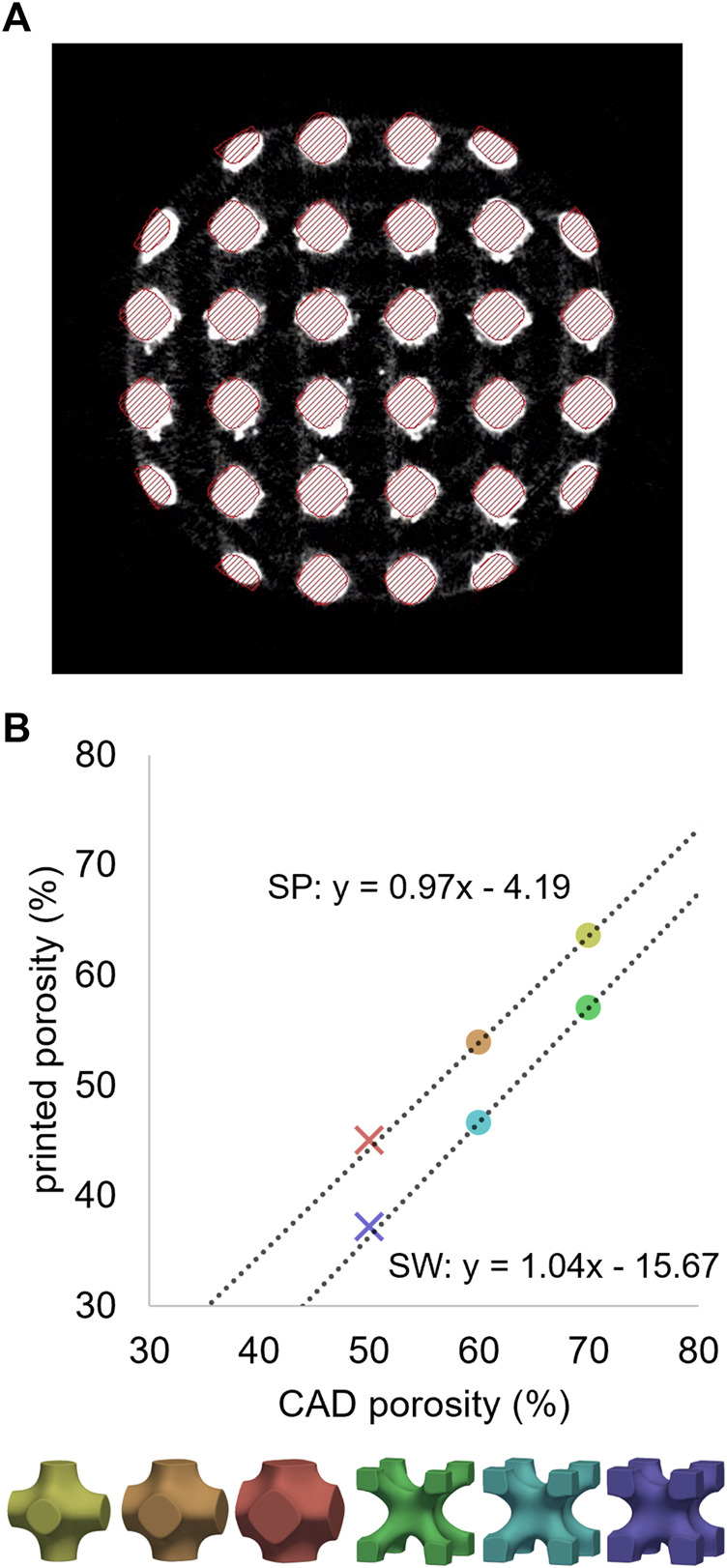
**(A)** µCT cross-section of a 60SP sample in grayscales with the CAD model overlayed with red hatches. **(B)** Relationship between the porosities of the original CAD models and the 3D-printed samples determined by µCT imaging. Unit cell type-wise linear regressions performed on the calibration dataset (circles) provided accurate predictions for the validation designs (crosses).

For all six pore geometries, the µCT-based printed porosity was lower compared to the original CAD-based planned porosity due to additional material sintered during manufacturing (Figure 6A). This difference varied between the cell types, with approximately 4% of extra material for SP and 16% for SW designs. Cell-specific correlations were established based on the calibration dataset and were used to estimate the adjusted geometries of the validation designs. These predictions provided less than 1% error when compared to the µCT-based values of the validation specimens (Figure 6B), indicating consistent relationship for a given pore cell type. The adjusted versions of the CAD models were generated to match these µCT-based porosity magnitudes. For instance, an adjusted CAD model with 64% porosity was generated to replicate the printed porosity of design 70SP.

### 3.2 Mechanical testing

#### 3.2.1 Monotonic testing

**FIGURE 7:**
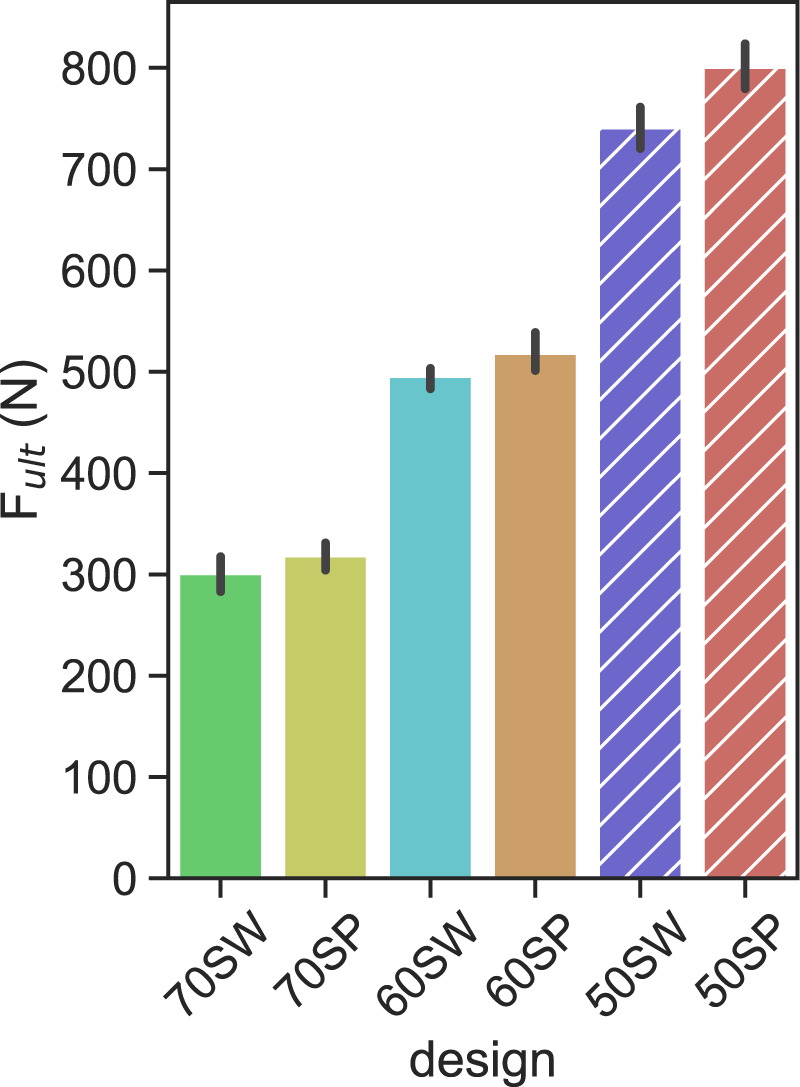
Ultimate load levels measured for each porous configuration. the solid and hatched bars indicate the calibration and validation designs, respectively. Error bars indicate 95% confidence interval.

The monotonic ultimate load results showed strong dependency on porosity, with a 10% decrease of porosity corresponding to an approximately 200 N increase of ultimate load (Figure 7). These trends were similar for the two different unit cell types SP and SW.

#### 3.2.2 Fatigue testing

**FIGURE 8:**
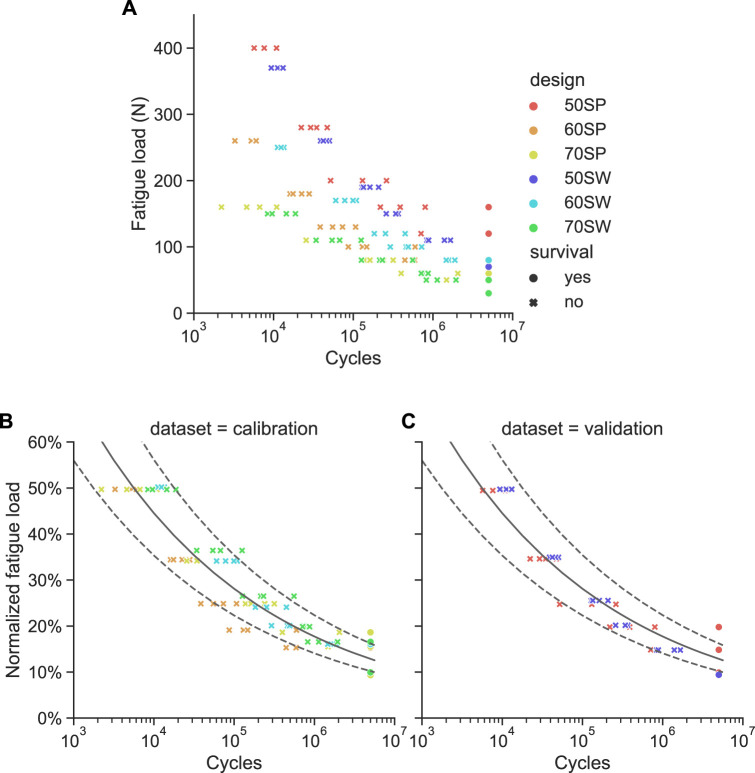
Results of the fatigue failure tests. **(A)** Absolute fatigue load magnitude *versus* cycles to failure for all six pore configurations. **(B)** and **(C)** Same data with the fatigue load normalized to the monotonic ultimate load for the calibration **(B)** and validation **(C)** sample sets. The power law fitted on the calibration dataset is represented as a solid line with the dotted lines being its 95% confidence interval.

The absolute fatigue load magnitudes plotted against the number of cycles to failure showed a wide spread for the different designs (Figure 8A), confirming the validity of the underlying assumption. This scatter was considerably reduced when normalizing the fatigue load magnitudes by the monotonic ultimate load values (Figure 8B). For each design, all four samples of a given load level survived at the 10% of $F_{ult}^{\exp}$ while at least one of the four specimens failed at the 15% of $F_{ult}^{\exp}$ . The normalized endurance limit is therefore comprised in the 10%–15% interval. Fitting Eq. 1 to the samples of the calibration sets with $N_{f}$ < 5 million cycles provided a = 3.22 and b = −0.20. This relationship was found to be consistent with the results of the validation set (Figure 8C), corroborating the appropriateness of this approach and the determined parameters. The endurance limit at 5 million cycles estimated by this relationship was equal to 12.86% of $F_{ult}^{\exp}$ , with a 95% confidence interval of [10.06%, 16.43%]. These values were consistent with the 10%–15% interval determined based on survivals. The sample survivals of the validation set were also within this confidence interval (Figure 8C).

#### 3.2.3 Uniaxial tensile testing

The following elastic and plastic material properties of the 3D-printed titanium were determined by uniaxial tensile testing of dog bone shaped specimens: E = 104.8 GPa (standard deviation (SD): 3.7 GPa), σ_y_ = 820.6 MPa (SD: 6.0 MPa), UTS = 914.6 MPa (SD: 16.3 MPa) and A% = 4.41% (SD: 1.33%). The mean values of these properties were used as material parameter input in the FE models.

### 3.3 FE simulations

**FIGURE 9:**
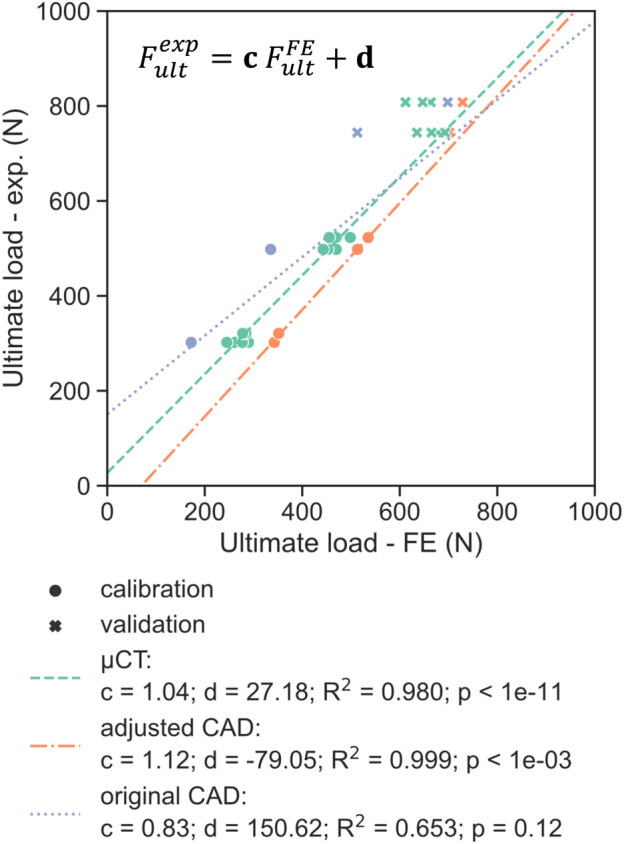
Comparison of the experimental ultimate load with the FE-predicted ultimate load for the three different model geometries (µCT, adjusted CAD and original CAD), with the latter determined using a plastic displacement offset set at 0.07 mm. The dotted lines represent the linear regression determined on the calibration dataset (circles), indicating good fit for the validation set (crosses) for the adjusted CAD approach.

The best correlation (R^2^ = 0.98) between experimental monotonic ultimate load and FE-predicted ultimate load using the µCT-based models was found for $u_{ult}^{pl}$ = 0.07 mm. Using this evaluation offset value, a high correlation was observed also for the adjusted CAD-based models (R^2^ = 0.99). These two model types possessed significant correlations (*p* < 0.001), contrarily to the original non-adjusted CAD-based ones (*p* = 0.12 and R^2^ = 0.65, Figure 9). Using this approach, good prediction of the experimental monotonic ultimate load could be achieved for the validation set (Figure 9).

### 3.4 Endurance limit prediction

**FIGURE 10:**
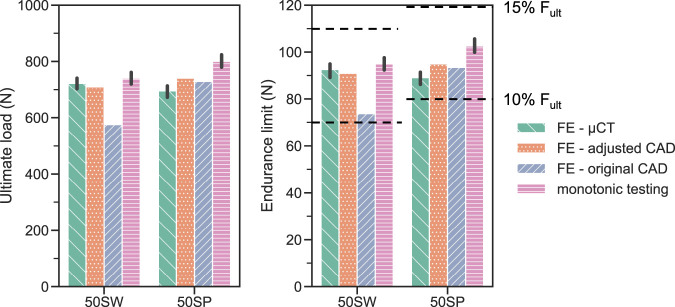
FE-based predictions of monotonic ultimate load (left) and fatigue endurance limit (right) compared with the corrsponding experimental results (pink bar: monotonic ultimate load; dotted lines: endurance limit, comprised between 10% and 15% of $F_{ult}$ ). The error bars represent the 95% confidence intervals computed either on the four samples tested monotonically (monotonic testing) or on the four µCT-based FE results (FE - µCT).

For both validation designs 50SW and 50 SP, the FE-based endurance limit predictions were within the 10%–15% $F_{ult}$ range determined experimentally (Figure 10), confirming the validity of the developed and calibrated simulation workflow. Since the specific aim of FE modeling in this workflow was to predict the ultimate load, its accuracy must be assessed relatively to the experimental monotonic test results. In this regard, models with the adjusted CAD-based geometry provided the most consistent estimations of experimental monotonic ultimate load for the two designs of the validation set (50SP and 50SW) with a maximum relative error of 8%. In comparison, the maximum error of the models with the original CAD based and µCT geometry were 23% and 15.3%, respectively.

## 4 Discussion

The FE simulation approach developed and calibrated in this study was found to accurately predict an implant design’s infinite fatigue life for a validation set having different porous configurations. This prediction tool can be an efficient surrogate for time consuming fatigue testing directly based on CAD models and therefore accelerate the development process of porous implants. This approach can also be utilized as a benchmark to evaluate the fatigue strength of different porous configurations under bending-compression loading corresponding to the ISO 14081 standard.

Another finding of this study was that monotonic testing appeared to be a good surrogate of fatigue testing. The normalized fatigue life curves (Figures 8B,C) were consistent for all designs and, most importantly, the endurance limits were reached at the same normalized load level range of 10%–15% of $F_{ult}$ . From an implant development perspective, this result shows the potential of monotonic testing to be used for rapid screening of several designs, enabling the selection of the most promising designs to be tested cyclically, reducing the number of time-consuming fatigue tests.

The normalized endurance limit determined in this work was significantly lower than that of solid titanium ( $\sigma_{e}/\sigma_{y}$ ≈ 40%) (Amin Yavari et al., 2013). This discrepancy was most probably caused by manufacturing imperfections such as surface roughness and irregular strut thicknesses where fatigue crack initiation is more likely to occur (Dallago et al., 2018; Benedetti et al., 2021). Several studies reported that porous titanium structures have a lower fatigue resistance than solid titanium. Vanmunster et al. tested a porous dental implant under ISO 14801 conditions and reported an endurance limit equal to 14.7% of $F_{ult}$ (Vanmunster et al., 2022). Both Lietaert et al. and Amin Yavari et al. determined the S-N curves of porous cylinders under uniaxial compressive loading (Amin Yavari et al., 2013; Lietaert, 2018). Extrapolating the fatigue failure relationships reported by them to 5 million cycles results in fatigue limits ( $\sigma_{e}$ ) of 7.7% and 16.4% of $\sigma_{y}$ , respectively. These values are consistent with the normalized endurance limit determined in the present work.

The fact that the experimental ultimate monotonic load was found in this study to be a good surrogate of endurance limit supports our initial choice to simulate the monotonic test with the FE models. With this approach, only one parameter ( $u_{ult}^{pl}$ ) was needed to be calibrated experimentally. Modeling the fatigue behavior would have presented the advantage of establishing a direct link between the simulations and the experimental fatigue behavior. The feasibility of this approach was demonstrated by the implementation of fatigue algorithms for porous cylinders (Hedayati et al., 2016; Zargarian et al., 2016) and conventional dental implants (Tsai et al., 2013; Prados-Privado et al., 2018; Lee et al., 2021). However, implementing this was beyond the scope of the present study since, among other challenges, characterizing the fatigue properties of the printed material would have been necessary and the complexity of the model would have increased. Similarly to the present study (Wang et al., 2019), combined ISO 14801 experimental testing of a porous dental implant with FE modelling. They simulated an implant integrated in a mandibular cross-section and loaded monotonically 30° off-axis. This configuration did not reproduce accurately the experimental test but allowed a qualitative comparison, with their porous implant showing higher stress levels *in silico* and a shorter fatigue life *in vitro* than the solid one.

Three strategies that were employed in this study to define the FE geometry for each design. Utilizing the original CAD design is generally the most convenient approach since it does not require any additional characterization of the printed samples. Hence, most *in silico* studies modelled porous dental implants with as-designed CAD geometries (Wang et al., 2019; Huang et al., 2020; Zhang et al., 2020). The ultimate loads computed with this method, however, did not correlate significantly (*p* = 0.12) with the experimental results (Figure 9). In addition, the worst prediction was achieved with this geometry during model validation, with the ultimate load of 50SW being underestimated by 23% (Figure 10). This discrepancy may be explained by the different printability of the two unit cell types. The µCT analysis revealed that the printed porosities were lower than for the CAD-based planned geometries, by approximately 4% for SP and 16% for SW. For the same CAD-based planned porosity, $F_{ult}^{\exp}$ was higher for SP than for SW (Figure 7), confirming the contribution of the extra printed material to the mechanical strength of the resulting. porosity shows that SP has a higher strength. Thus, 50SP and 60SW have similar printed porosities (Figures 6–b) but the ultimate load of 50SP is superior by 60%.

The µCT-based modeling approach provided predictions that correlated well with experimental data (R^2^ = 0.98) since it incorporated the real geometry of the samples. Simulating four scanned samples per design showed reasonably small variability of the estimated ultimate loads (Figure 9). These models took into account manufacturing irregularities that reduce the mechanical strength of porous titanium scaffolds as highlighted by Liu et al. (2017). For example, stress concentrations can be caused by an irregular strut thickness, while aggregates of partially fused particles on the surface contribute less to structural stiffness (Figure 6A). However, this approach cannot be used in the optimization workflow in a prospective manner aiming to predict the endurance limit of new porous configurations without the necessity of manufacturing. Adjusting the CAD geometry provided a good compromise between the exact representation of the printed geometry and a CAD-generated geometry that is suitable for the purposes of a numerical predictive workflow. For all designs, adjusted CAD-based FE models exhibited higher ultimate loads than µCT-based ones, although these should be similar in the case of an ideal adjustment. This difference is most likely caused by the lack of irregularities of the adjusted CAD geometry. This optimal repartition of the same volume of material results in a higher predicted monotonic strength.

This study has a number of limitations that should be addressed. First, this workflow was able to predict the endurance limit for porous configuration variations, but its validity was not assessed for other design features such as the overall dimensions of the implant (diameter, length) or the position and shape of the porous region. The fatigue strength of an implant could, for example be enhanced by adding solid elements to stiffen structure. Such approach was adopted by (Xiong et al., 2020), who optimized the strength of a porous implant in uniaxial compression by varying the diameter of a solid central rod. Adapting the method developed here to a full implant design may require a partial recalibration of some parameters, e.g., the plastic failure displacement. The monotonic failure criterion was defined here based on displacement and not stress. A stress-based failure criterion would be independent from the global sample geometry and would consist in predicting strut failure. However, Benedetti et al. reported that the failure mode of a porous structure was dependent of unit cell topology and can result in different degrees of ductility (Benedetti et al., 2021). Lastly, the scope of ISO 14801 limits the pore locations that can be investigated with the current method. The part of the sample starting 3 mm below nominal bone level and therefore all implant features below the embedding plane are not considered in these tests. Since the purpose of porous dental implants is primarily to increase osseointegration, many designs reported in the literature (Obaton et al., 2017; Yang et al., 2017; Hong et al., 2020) incorporate pores located in this “blind spot”. As such, alternative methods have been employed to test these designs, either experimentally (e.g., three point bending, uniaxial compression) (Chakraborty et al., 2020; Huang et al., 2020; Xiong et al., 2020), or numerically (e.g., simulating the implant’s behavior in bone) (Liu et al., 2017; Zhang et al., 2020).

In conclusion, a fatigue life prediction tool for porous dental implants was developed, calibrated, and validated in the present study possible by combining *in vitro* and *in silico* methods. Fatigue and monotonic mechanical testing were performed on six porous configurations of a simplified implant design. The monotonic failure load was found to be a good and efficient surrogate of the fatigue failure load, with the endurance limit of all designs being situated between 10% and 15% of the monotonic ultimate load. FE simulations predicted well the experimental monotonic test results when the model geometry was based on µCT images, but not for as-designed CAD-based model geometries. This approach ignores the discrepancies between CAD and printed geometries. This limitation was resolved by utilizing CT-based geometry or by adjusting the porosity of the CAD model.

## Data Availability

The raw data supporting the conclusion of this article will be made available by the authors, without undue reservation, upon specific request.
